# Different electrode positioning for transcutaneous electrical nerve stimulation in the treatment of urgency in women: a study protocol for a randomized controlled clinical trial

**DOI:** 10.1186/s13063-020-4096-7

**Published:** 2020-02-11

**Authors:** Juliana Falcão Padilha, Mariana Arias Avila, Enio Júnior Seidel, Patricia Driusso

**Affiliations:** 1 Physical Therapy Department, Federal University of Sao Carlos (UFSCar), Rodovia Washington Luis Km 235, São Carlos, São Paulo CEP 13565-905 Brazil; 2 Department of Statistics, Federal University of Santa Maria (UFSM), Av Roraima 1000, Santa Maria, Rio Grande do Sul CEP 97105-900 Brazil

**Keywords:** Overactive urinary bladder, Lower urinary tract symptoms, Tibial nerve, Electric stimulation therapy

## Abstract

**Background:**

Urgency is a complaint of sudden, compelling desire to pass urine, which is difficult to defer, caused by involuntary contraction of the detrusor muscle during the bladder-filling stage. To enable detrusor inhibition, electrotherapy resources such as transcutaneous tibial nerve stimulation (TTNS) and parasacral transcutaneous electrical stimulation (PTES) have been used. The objective this study is to publish the study protocol that aims to investigate whether urgency decreases after treatment with both of the techniques.

**Methods:**

This randomized controlled clinical trial will include 99 women, aged more than 18 years old, with urgency (score ≥ 8 in the Overactive Bladder-Validated 8-Question Awareness Tool [OAB-V8]). Women will be randomly allocated into three groups: TTNS, PTES, and placebo. The following questionnaires will be applied: the Anamnesis Record, the Incontinence Questionnaire Overactive Bladder, the King’s Health Questionnaire, the 24-Hour Voiding Diary, and the OAB-V8, at four different time points: at baseline prior to the first session, at the 6th session, the 12th session and at follow-up. The current used for the transcutaneous electrical stimulation will be a symmetrical balanced biphasic pulsed current, for 12 sessions, twice a week, for 20 minutes. Qualitative variables will be displayed as frequency and percentage, quantitative variables as mean and standard deviation. Comparison of urgency severity among groups will be performed with a repeated measures ANOVA, considering the effect of the three groups and the four evaluations, and interactions among them.

**Discussion:**

The present study aims to contribute evidence for a more in-depth discussion on electrode positioning for electrostimulation used in urgency treatment. It should be emphasized that, based on the possibility of confirming the hypothesis that urgency will decrease in a similar way after both treatments (TTNS and PTES), the PTES will be used as an option for positioning the electrodes alternatively to the tibial nerve region in special populations, such as amputees or people with severe lower limb sensory impairment.

**Trial registration:**

Brazilian Registry of Clinical Trials (ReBEC) ID: RBR-9rf33n, date of registration: 17 May 2018.

## Introduction

The International Continence Society defines urinary incontinence (UI) as any involuntary loss of urine [1, 2] and is classified as urinary stress incontinence, urgency urinary incontinence (UUI), and mixed urinary incontinence [3]. UUI occurs due to a contraction of the detrusor muscle at the bladder filling stage, with urine loss, accompanied or preceded by urgency [4]. An overactive bladder (OAB) occurs when there are symptoms of urgency with or without UI, usually polyuria and nocturia [4, 5].

UI is a public health problem, affecting, above all, older people [6] and women [7]. With regard to UUI, it is associated with a significant and clinically important decrease in health-related quality of life (QoL) [8, 9]. The prevalence of UUI is 1.8–30.5% in European populations, 1.7–36.4% in the United States, and 1.5–15.2% in Asian populations [10].

Electrical stimulation is used as a resource for UUI treatment in the physiotherapist’s clinical practice [11, 12]. Transcutaneous tibial nerve stimulation (TTNS) aims to facilitate inhibition of the detrusor muscle [13] by means of electrostimulation in the path of the tibial nerve [14]. The tibial nerve is a mixed nerve [15], that originates in the L5-S3 nerve roots, which are the same roots that innervate the parasympathetic pathway of the bladder (S2-S4) [14, 16], thus, it is suggested that direct stimulation of the tibial nerve could inhibit the S2-S3 afferents, thereby decreasing detrusor hyperactivity. This is a simple, non-invasive, well-tolerated technique considered conservative and effective therapy for patients with UUI [17] and OAB [11].

Guidelines, such as the Canadian Urological Association Guideline on Adult Overactive Bladder (2017) [5], European Association of Urology Guideline in Urinary Incontinence in Adults (2018) [13], and the American Urological Association/Society of Urodynamics, Female Pelvic Medicine and Urogenital Reconstruction Guideline: Overactive Bladder in Adults (2019) [18] report TTNS as the third line of treatment for UUI, with level of evidence B [5, 13] and C [18]. A recent systematic review noted the lack of protocol standardization for this technique, such as parameters, number of sessions, and duration of treatment [19]. There is a need for further research to confirm the efficacy of specific subgroups of patients as well as the magnitude of effect sizes associated with the ideal stimulation program and duration of effect [19]. On the other hand, a prospective cohort study found significant symptomatic improvement in all clinical parameters after treatment with TTNS in volunteers with UUI, with no adverse effects during treatment [17]. However, there are still gaps in the literature, and therefore studies with more consistent methods are needed, which strengthens the development of this study protocol using TTNS.

Another electrotherapy technique used in clinical practice is parasacral transcutaneous electrical stimulation (PTES), which uses a low-frequency current through transcutaneous electrodes in the sacral region (S3), with the aim of promoting an inhibitory reflex for detrusor muscle inhibition [20], and a consequent reduction in urgency. PTES is used in the treatment of voiding disorders in children [21–23] and clinical trials have reported positive effects for OAB in children and adolescents [24–26]. Sharma et al. [27] found a reduction in nocturia, urgency, and urge-incontinence in women with OAB, treated with PTES. However, the literature lacks more robust clinical trials using this form of application in adult women as well as clinical trials comparing the efficacy of PTES and TTNS.

The general objective of the research is to investigate whether urgency will decrease after treatment with transcutaneous electrostimulation in the tibial nerve or in the parasacral region. The hypothesis of the study is that urgency will decrease similarly after both treatments. The purpose of this article is to describe the methods and statistical analysis of this study, so that this information can be made public.

## Methods

### Trial design

This is a blinded randomized controlled trial. Outcome measures will be assessed at baseline (prior to initiation of treatment), during treatment (at the 6th session), on the last day of treatment (the 12th session), and 1 month after the completion of treatment (follow-up). Starting on March 1, 2017, participants were enrolled and randomized into three groups (TTNS, PTES, and placebo electrostimulation) by simple randomization using a 1: 1: 1 ratio. Data for the primary analysis will be collected by December 2019 and follow-up data by February 2020. Currently, no data analysis has been performed and the allocation of the groups remains blinded to the evaluator and the participants. The planning of this project followed the guidelines of the Consolidated Standards of Reporting Trials (CONSORT) and Standard Protocol items: recommendations for interventional trials (SPIRIT) guidelines.

### Ethics

The research was approved by the Ethics and Research Committee of the Federal University of São Carlos (CAAE: 79893917.1.0000.5504). Each participant will receive clarification regarding the research objectives, anonymity of their data, and freedom to participate. Participants will sign two copies of the Informed Consent Term (ICT). This study will respect the ethical precepts of Resolution CNS 510/2016 and be performed according to the Declaration of Helsinki. This study was registered in the Brazilian Registry of Clinical Trials (ReBEC) under number http://www.ensaiosclinicos.gov.br/ ID: RBR-9rf33n, http://www.ensaiosclinicos.gov.br/rg/RBR-9rf33n/, date of registration: May 17, 2018.

### Participants and settings

The trial is underway in the Laboratory of Research in Women’s Health (LAMU), in the Physiotherapy Department of the Federal University of São Carlos (UFSCar), in São Carlos, Brazil. Participants are being invited through informal invitation in social groups, and the research will also be disseminated through the internet on the UFSCar dissemination site and on social networks and leaflets. The objectives of the study will be explained by the researcher, who will clarify all doubts and questions in relation to the research, and individuals who agree to participate will sign the ICT. After selection of the participants, scheduling will take place for evaluations of eligibility of the sample and, later, for the start of treatment.

The inclusion criteria considered will be: women over 18 years of age; with urgency, self-reported and confirmed by a score greater than or equal to 8 on the Overactive Bladder – Validated 8-Question Awareness Tool (OAB-V8) [28] questionnaire. The exclusion criteria will be: stress urinary incontinence with no urgency symptom; urinary infection; alcoholism, smoking, or drug addiction; lesions and alteration in cutaneous sensitivity in the place where the electrotherapy will be applied; cognitive deficits; drug and/or physiotherapeutic treatment for UUI or current OAB; having any neurological disease; being in the gestational or puerperium period.

The presence of any signs of skin irritation that may be associated with electrostimulation will be considered as the criterion to discontinue the treatment. No concomitant care and interventions will be encouraged or prohibited during the study.

### Sample size

The calculation of the sample size was performed in G*Power 3.1.9.2 software, considering the comparison of three independent groups (TTNS, PTES, and placebo electrostimulation) in four distinct stages (pretreatment, 6th and 12th evaluation, follow-up), an analysis of variance (ANOVA) for repeated measurements, effect size of 0.15, significance level of 5%, and 90% test power, resulting in a total of 99 participants in the study (33 participants per group).

### Randomization and allocation

**Fig. 1:**
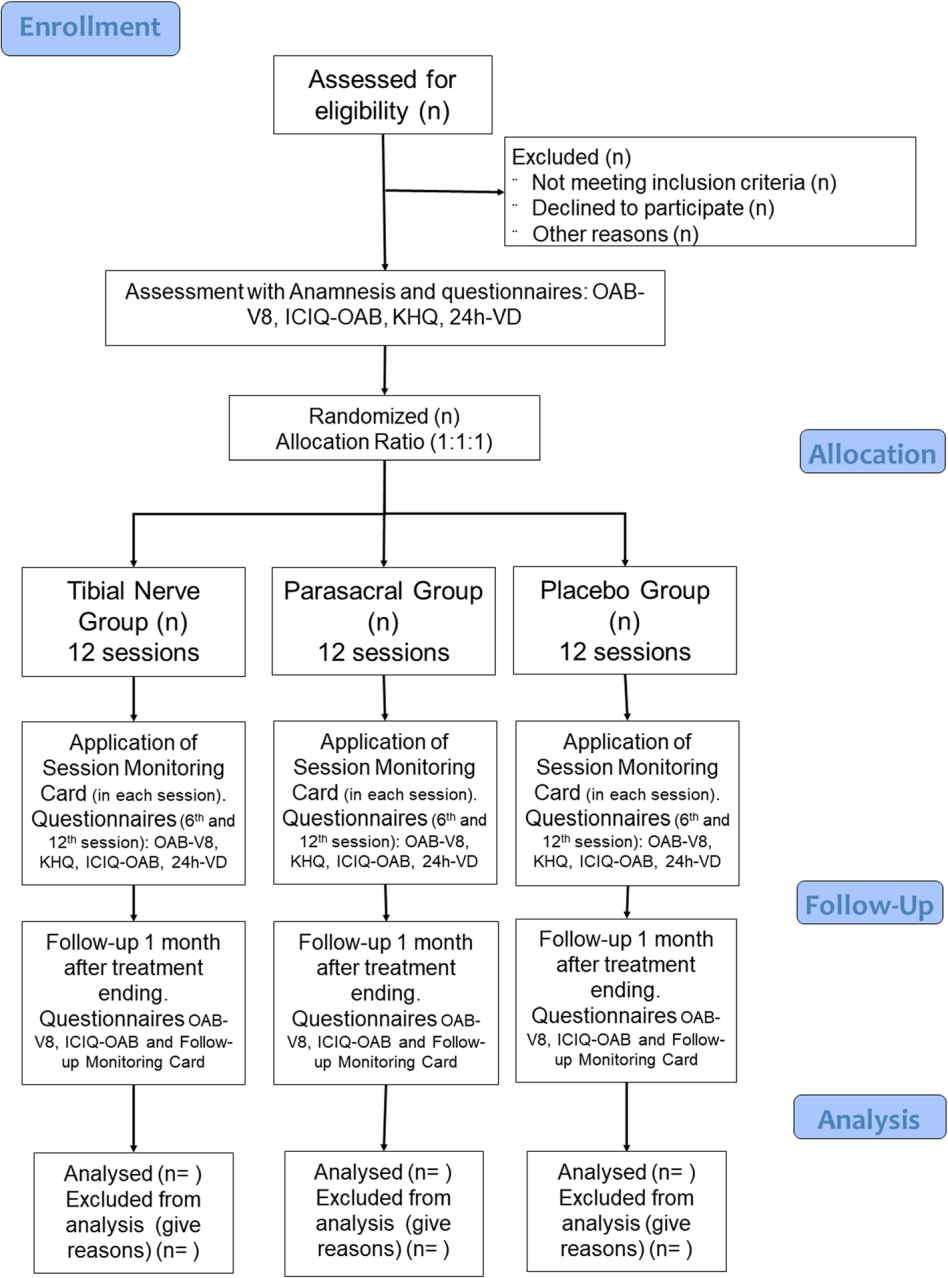
Trial flow diagram. *24 h-VD* 24-Hour Voiding Diary;; *ICQ-OAB* Incontinence Questionnaire Overactive Bladder; *KHQ* King’s Health Questionnaire; *OAB-V8* Overactive Bladder – Validated 8-Question Awareness Tool

Once included, initial evaluations will be conducted before participants are randomized (1: 1:1) to three groups (TTNS, PTES, and placebo electrostimulation, Fig. 1), using simple randomization, conducted by an investigator who is not involved with the recruitment and treatment of participants.

Ninety-nine pieces of paper cut in rectangular form in three distinct colors, corresponding to TTNS, PTES, and placebo, respectively, will be placed inside a brown envelope. The volunteer will remove 1 (one) piece of colored paper from the envelope and then show it to the blind evaluator, who will record the color of the treatment drawn.

On the first day of treatment the physiotherapist will check the color chosen and apply the corresponding technique. This physiotherapist will not be involved in the randomization, evaluations, or re-evaluations of the participants. Participants will be informed that they will receive one of three different forms of application of electrostimulation, but will be blinded to the hypothesis of the study.

### Evaluation tools

After the participants sign the ICT, an investigator will apply validated questionnaires that measure the severity of the OAB (Overactive Bladder – Validated 8- Question Awareness Tool; OAB-V8) and UUI (Incontinence Questionnaire Overactive Bladder; ICIQ-OAB), and quality of life, specifically the history of urgency and urinary incontinence (King’s Health Questionnaire; KHQ). The participant will be given a 24 hour Voiding Diary (24 h-VD) (the participant will be asked to deliver the completed diary on the first day of treatment). In the 6th and 12th sessions, these four questionnaires will again be completed as a way of evaluating the course of treatment, by the same evaluator blind in relation to the groups (Table 1).

**Table 1:** Data collection/measurement of results

	Pretreatment	1st Session	2nd Session	3rd Session	4th Session	5th Session	6th Session	7th Session	8th Session	9th Session	10th Session	12th Session	Follow-Up
Anamnesis record	+												
OAB-V8	+						+					+	+
ICIQ-OAB	+						+					+	+
KHQ	+						+					+	
24 h-VD	+						+					+	
Session Monitoring Card		+	+	+	+	+	+	+	+	+	+	+	
Follow-up Monitoring Card													*+*

*24 h-VD* 24-Hour Voiding Diary; *ICIQ-OAB* Incontinence Questionnaire Overactive Bladder; *KHQ* King’s Health Questionnaire; *OAB-V8* Overactive Bladder – Validated 8-Question Awareness Tool

The OAB-V8 is an instrument translated and validated for Portuguese [28]. It includes eight questions alluding to an initial general statement “How much have you been bothered by ...”. As options for answers, there are six variations “Never; Almost nothing; A little; Enough; A lot, and Very Much”. The score of this instrument is calculated through the sum of the questions, and can vary from 0 to 40 points; if the result is 8 or more than 8, it is indicative of OAB [28]. The ICIQ-OAB has been translated, adapted, and validated into Portuguese [29]. This is a short questionnaire with a high psychometric capacity to evaluate OAB, capable of assessing the impact of symptoms of urinary frequency, urgency, nocturia, and incontinence [30]. It consists of four basic questions: question 3a investigates urinary frequency, question 4a evaluates nocturia, and questions 5a and 6a investigate urgency and UUI, respectively. For the analysis of the results, the corresponding values of questions 3a, 4a, 5a, and 6a are added, obtaining a total of 0 to 16 points; the higher the value found, the greater the impairment [29].

The KHQ has been translated and validated into the Portuguese language [31]. This questionnaire is specific to UI [32]. The KHQ consists of 30 questions, divided into eight domains: general health perception, impact of UI, limitations of daily activities, physical limitations, social limitations, personal relationships, emotions, sleep/mood. The questionnaire also contains two other independent scales: one assesses the severity of UI (severity measures) and the other the presence and intensity of urinary symptoms (urinary symptom scale). The KHQ is scored in domains, so there is no overall score. The scores range from 0 to 100 and the higher the score obtained, the worse the quality of life related to that domain [31].

The 24 h-VD [3], contains data on urinary habits, and should be completed for a period of 24 hours, with information on number of urinations; amount of urine eliminated; and occurrence of urinary loss or urgency. During the study, the participant will complete the diary 3 times, referring to pretreatment, middle of treatment (6th session), and end of treatment (12th session).

During the treatment, in order to follow the micturition symptoms per session, a Session Monitoring Card will be used. This fact sheet contains open and closed questions about the urinary symptoms of the day before the session and the day of the session. It also contains a Visual Analogue Scale (VAS) [33] to indicate possible discomfort of the applied technique. The volunteer will be questioned as to their degree of discomfort during the treatment, with 0 indicating total absence and 10 the maximum level of discomfort bearable. Any side effects that the participants report will be recorded by the researcher. Over the 12 sessions, each participant will complete 12 Session Monitoring Cards, thus allowing longitudinal analysis of the voiding symptoms.

One month after completion of treatment, an investigator, blinded to the allocation of participants, will complete a Follow-up Monitoring Card. In addition to this card, the ICIQ-OAB and OAB-V8 will also be applied.

### Interventions

A total of 12 sessions of electrostimulation treatment will be performed, twice a week, on non-consecutive days. In order to minimize any effects of physiological filling of the bladder due to the applied technique, as part of the intervention procedure, the participants will be required to try to empty the bladder before each session, even if they report the absence of the need to urinate. In order to verify the post-void residual volume, an ultrasound (US) examination will be performed using a GE Healthcare Venue 40® ultrasound (probe 4C) (GE Healthcare, Chicago, IL, USA). The participant will remain lying on a bed in the supine position, the infra-abdominal region will be cleaned with 70% alcohol, with the ultrasound conducting head positioned vertically on the lower abdomen to locate the bladder and perform the necessary measurements. The following equation will be used to measure bladder volume: Volume = Length × Width × Height × 0.52, this being an easy, fast, and reliable method [34]. The post-void residual volume will be considered as values of up to 50 ml [35]. As a way of controlling the volume of urine, the same US procedure will be performed after the intervention (treatment). This procedure will be performed pre- and post-intervention treatment in all sessions of electrostimulation.

The electrical stimulation techniques used will be TTNS, PTES, and placebo electrostimulation. During the treatment all sessions will be conducted according to the following flowchart (Fig. 1) for the three groups.

**Fig. 2:**
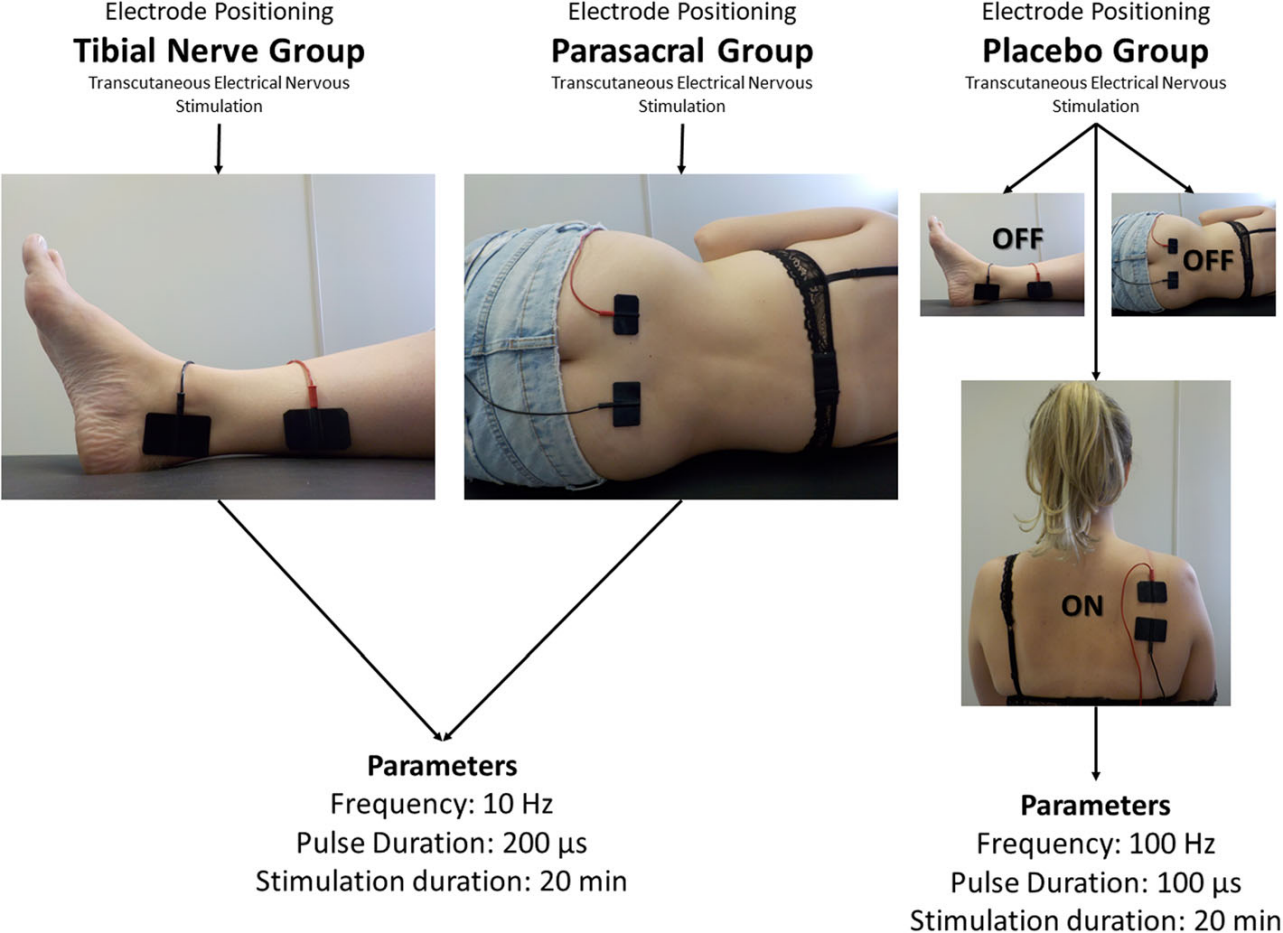
Layout of the positioning of the electrodes and parameters for each group

For the description of the correct way to report electrostimulation, the recommendations of Barbosa et al. [36] will be followed. For the treatment with electrostimulation, a balanced asymmetric pulsed current of constant voltage will be used, in transcutaneous application mode, with a Tens Vif 993 Four four-channel device (Quark®, Brazil). The company that produces the device has the proper credentials in the Registry with the National Health Surveillance Agency (ANVISA) under registration number 80079190023. The company meets the standards NBR ISO 9001, NBR ISO 13485 and RDC 59 (Good Practice Guidelines) and has the authorization of the Brazilian Health Ministry. The electrodes will be surface (silicone rubber and carbonate), brand Quark®, with dimensions of 5 cm by 3 cm, coupled with conductive gel and fixed on the skin of the volunteer with micropore tape. The positioning of electrodes and adopted parameters can be observed in Fig. 2.

The same parameters will be used in both groups, PTES and TTNS: frequency 10 Hz (Hertz), pulse duration 200 μs (microseconds), during 20 minutes [25, 37]. Every 3 minutes the participant will be asked about the sensation and, if necessary, the intensity will be increased, always maintaining a strong but not painful sensation.

For the positioning for the TTNS technique, the volunteer will be placed in the supine position on the bed with knee in semi-flexion and head resting on a pillow. The electrodes will be positioned one immediately posterior to the medial malleolus of the ankle and another approximately 10 cm above this, fixed with micropore tape. In order to ensure that the electrodes stimulate the tibial nerve, a current with a frequency of 1 Hz will be applied first, with a gradual increase in intensity to verify a rhythmic flexion movement of the hallux. After confirming the correct positioning, the device will be switched off and when the device is switched on again, the above parameters will be used and the initial intensity adjusted according to the sensitivity of the participant, below the motor threshold [37].

For the PTES technique, the participant will be positioned in left lateral decubitus on the bed, with 90° hip flexion, knee in semi-flexion, erect trunk, and head resting on a pillow. The electrodes will be placed side by side symmetrically, with conductive gel, approximately four centimeters apart, at the level of the third sacral vertebrae (S3). For the exact positioning of the electrodes, the anatomic reference bone, posterior superior iliac spine (PSIS) (which corresponds to the level of the second sacral vertebrae [S2]) will first be located, after which the electrodes will be positioned using the measurement of the participant’s two-finger distance below the PSIS.

Regarding the placebo group, the participant will be positioned in left lateral decubitus on the bed with 90° hip flexion, knee in semi-flexion, erect trunk, and head resting on a pillow. The electrodes will be placed concomitantly at three sites: the tibial and parasacral positionings (mentioned above), and the scapula, with an inter-electrode distance of approximately 4 cm; however, only the channel to the scapular region will be activated, the rest remaining inactive (adapted from Lordêlo et al. [25]). It should be noted that the region of the scapula is not part of the nerve path referring to the detrusor, or pelvic floor, thus designated as no effect for treatment of voiding dysfunctions. The parameters used will be a frequency of 100 Hz, pulse duration 100 μs, and time of 20 minutes. This configuration is known as conventional transcutaneous electrical nerve stimulation [38], and commonly used for non-invasive and non-pharmacological treatment of pain, free of side effects [39, 40]. Monitoring of intensity will be the same as for the electrostimulation protocols described above.

### Blinding

Given the nature of the researcher, it is not possible to blind participants to electrostimulation. However, the participants are blinded to the hypothesis of the study and to the group in which they are allocated. The principal investigator, due to the nature of the intervention, cannot be blinded in relation to the groups, but will be blinded on the outcomes of the evaluations made by the assessor blinded to the participants’ allocation.

### Measurement of results, primary and secondary outcomes

The outcome measures will be evaluated in several stages: at the beginning before the randomization (Anamnesis record, OAB-V8, ICIQ-OAB, KHQ, 24 h-VD), during the 12 sessions (Session Monitoring Card), in the 6th and 12th sessions (OAB-V8, ICIQ-OAB, KHQ, 24 h-VD), and 30 days after the end of treatment (Follow-up Monitoring Card, OAB-V8, ICIQ-OAB) (Table 1). The primary outcome of the study is urgency, measured by the OAB-V8. This was chosen as it is a validated instrument for Portuguese, with criteria defined for identification of urgency, easy to apply, objective, with robust results. It is used both as a screening tool and to raise self-identification awareness of the participant. In addition, as a primary outcome, the number of episodes of urgency will be observed (obtained in the Session Monitoring and Follow-up sheets). As secondary outcomes: voiding habits (number of diurnal urinations (Session Monitoring and Follow-up sheets and ICQ-OAB), use of absorbent pads (KHQ), urinary incontinence (number of episodes of urinary loss Session Monitoring and Follow-up sheets and ICIQ-OAB, severity of UI (ICIQ-OAB)) and quality of life (KHQ and ICIQ-OAB).

### Minimizing missing data

In order to avoid and minimize missing data, all participants receive paper reminders with the date and time of each session. The investigator also makes phone calls to reinforce the scheduling of sessions. For data from the 24 h-VD, participants are given reminders in previous sessions so that they will not forget to complete the diary. Regarding the follow-up, the blinded evaluator contacts the participant by telephone about 3 days before the scheduled date, in order to arrange the best time and date for the assessment to be performed. As a way of monitoring adherence to treatment, the attendance records will be registries in the Session Monitoring sheets. In cases of dropout or withdrawal from the study, the data will be analyzed by intention-to-treat analysis. Participants that leave the study without performing the 12 proposed sessions (due to sickness, moving to another town, inability to attend sessions) will be considered protocol deviations.

### Data management

The data are being collected in paper form and stored in binders in an allocated cabinet in the LAMU, UFSCar. After collection, all forms will be checked for data quality and missing values and will be stored in a locked cabinet to which only the principal investigator has access. The data will be entered in Excel 2011 software by the lead researcher. A standard coding manual describing the input data has been developed to ensure comprehension of the analysis.

The database and electronic analyzes will be stored on a secure computer server with personal login access authorized by the principal investigator. The principal investigator will have access to the complete data set (blind to group allocation) and the co-investigators will have access when necessary. After completion of the study, all data and study documents will be archived by the principal investigator and stored for 5 years in the Physiotherapy Department, UFSCar.

### Data monitoring

Researchers involved in the present investigation will be jointly responsible for the monitoring of the protocol along with relevant alterations that may occur during the development of the study. The University Post-Graduate Program will supervise for the integrity of the data, and the responsible Internal Data Monitoring Committee will have access to the patient allocation, while the whole analysis will be confidential.

### Harms auditing

All the medical records of the volunteer will be carefully assessed, and all harms and complications of the treatment will be reported when reporting the results of this trial, if any. The harms will be categorized as serious and minor adverse events.

### Auditing

The University Post-Graduate Program will supervise the integrity of the data.

### Statistical analysis plan

The statistical analysis will be performed by a statistician blind to the allocation of the groups and without involvement in the research. The software SPSS 16.0 and R 3.5.1 will be used for the analysis of data (IBM SPSS Statistics, Armonk, NY, USA). The level of significance will be set at 5%. The normality of the residues will be verified by the Shapiro-Wilk test.

The comparison of urgency, voiding habits, UI, and quality of life between groups (TTNS, PTES, and placebo) and between stages (pretreatment, 6th and 12th evaluation, follow-up), as well as the interactions between these will be performed by means of ANOVA of mixed models considering group and time factors for repeated measures if the residuals present normality with post hoc contrasts (treated versus placebo group, TTNS versus PTES).

The first statistical procedure will be to verify, in general and at each evaluation stage (pretreatment, 6th and 12th evaluation, follow-up) if the urgency, voiding habits, UI, and quality of life differ in the TTNS and PTES groups compared to the placebo group. The null hypothesis is that in both the TTNS group and the PTES group, urgency, urinary habits, urinary incontinence, and quality of life will be the same as the placebo group. The alternative hypothesis is that the TTNS and PTES groups will differ from the placebo group.

The second statistical procedure has the intention of verifying whether, in general and at each stage of evaluation (pretreatment, 6th and 12th evaluation, follow-up), urgency, voiding habits, UI, and quality of life differ between the TTNS group and the PTES group. The null hypothesis is that in both the TTNS group and the PTES group the behavior is the same. The alternative hypothesis is that there is a difference in behavior between these two groups.

The third statistical procedure aims to verify if there is alteration in the urgency, voiding habits, UI, and quality of life throughout the four stages of study evaluation (pretreatment, 6th and 12th evaluation, follow-up) within each of the groups. The null hypothesis is that the behavior is the same throughout the stages in each group. The alternative hypothesis is that there is a difference in the stages in each group.

The fourth statistical procedure is to verify if there is a change in the urgency, voiding habits, and IU throughout the 13 study evaluation stages (being the 12 session monitoring cards and 1 follow-up monitoring card) within each of the groups. The null hypothesis is that the behavior is the same throughout all stages in each group. The alternative hypothesis is that there is a difference throughout the stages in each group.

The quantitative variables will be presented as minimum value, maximum value, mean, median, standard deviation, coefficient of variation, and asymmetry coefficient. Categorical variables will be presented as frequencies and percentages.

Details of the statistical analyses are shown below:

1. In the interview, the anamnesis record will be applied, which generates qualitative variables that will be presented as frequencies and percentages, and quantitative variables that will be presented as minimum value, maximum value, mean, median, standard deviation, coefficient of variation, and asymmetry coefficient. The ICIQ-OAB, KHQ, OAB-V8, and 24 h-VD will be applied, which will result in quantitative scales that will be presented as minimum value, maximum value, mean, median, standard deviation, coefficient of variation, and asymmetry coefficient.
2. In the 6th and 12th sessions, the ICIQ-OAB, KHQ, OAB-V8, and 24 h-VD will be applied, which will result in quantitative scales that will be presented as minimum value, maximum value, mean, median, standard deviation, coefficient of variation, and coefficient of asymmetry.
3. In the follow-up, the ICIQ-OAB, OAB-V8 and Follow-up Monitoring Card will be applied, which will result in quantitative scales that will be presented as minimum value, maximum value, mean, median, standard deviation, coefficient of variation, and coefficient of asymmetry.
4. In all sessions during the treatment, a Session Monitoring Card will be applied that will generate quantitative variables that will be presented as minimum value, maximum value, mean, median, standard deviation, coefficient of variation, and coefficient of asymmetry.
5. At the end of the treatment, the ANOVA for repeated measurements will be applied to verify the effects of the three groups and the three stages (pretreatment, 6th and 12th evaluation), and the interactions between them in the KHQ, ICIQ-OAB, OAB- V8, and 24 h-VD, if the residues present normality. If the residues do not present normality, a non-parametric test will be applied.
6. At the end of the sessions and follow-up, the ANOVA for repeated measures will be applied to verify the effects of the three groups and the four stages, and the interactions between them in the OAB-V8, ICIQ-OAB, and Monitoring Cards (sessions and follow-up), if the residues present normality. If the residues do not present normality, a non-parametric test will be applied.
7. At the end of the sessions and the follow-up, the ANOVA for repeated measurements will be applied to verify the effects of the 13 stages (12 sessions and follow-up) of the Monitoring Cards (sessions and follow-up), if the residues present normality. If the residues do not present normality, a non-parametric test will be applied.

## Discussion

This study will demonstrate whether urgency decreases after treatment with transcutaneous nerve stimulation in the tibial nerve or the parasacral region. This will be observed in possible changes in the urgency symptom at the 6th and 12th sessions, or 1 month after the end of treatment using the OAB-V8, ICIQ-OAB, and Monitoring Cards. This study obtains a score of 8 in 10 on the Pedro scale, due to the impossibility of blinding the therapist and the participant (limitation inherent in the applied therapy). Up to the present moment, the study complies with 4 of the 6 items on this scale [41].

With regard to the electrotherapy, TTNS is a therapeutic treatment with evidence level B for the treatment of UUI and OAB. On the other hand, studies on PTES have been promising in the treatment of OAB Syndrome in children and adolescents. Both techniques are characterized as simple, non-invasive, and without side effects, which facilitates the acceptance of therapy by the women who need assistance. Although PTES therapy has shown good results, these are restricted to only a young age group, evidencing the paucity of studies in adult and elderly women who could benefit from the same technique. The addition of the placebo group will allow us to evaluate the true efficacy of electrical stimulation, be it TTNS or PTES.

According to a systematic review [42] on scientific evidence of tibial nerve stimulation in individuals with OAB, one of the main findings was the poor methodological quality of the studies, which may interfere with its reliability and application in clinical practice, although the majority of sample numbers were considerable. The same authors [42] further discuss the need for better designed clinical trials on this therapy, which further strengthens the present investigation with this protocol.

Based on this, the present protocol may corroborate with the literature on the effect of transcutaneous electrostimulation on urgency at the tibial nerve level and the parasacral region. Slovak et al. [14] in their study conclude that there is limited evidence of stimulation in other locations, the most logical approach to be used in transcutaneous electrical stimulation techniques seems to be stimulation in the sacral or tibial region, since they directly or indirectly target the medullar root S3.

In the protocol of the present study, due to the nature of the applied therapy, it is not possible to blind the physiotherapist as well as the participants. Ussing et al. [43], in their protocol, discuss the difficulty of blinding participants and health professionals when examining a physiotherapeutic intervention. In the study of these authors, in a similar way to the present study, the participants were blinded in relation to the hypothesis of the study. Participants are not informed, either verbally or in writing, that we expect electrostimulation to be more or less efficient between groups.

It should be emphasized that, based on the possibility of confirming the hypothesis that urgency will decrease in a similar way after both treatments (TTNS and PTES), the PTES will be used as an option for positioning the electrodes alternately to the tibial nerve region. Since people may have problems in the tibial nerve region such as sensitivity and skin changes, bilateral amputations, and in cases in which the patient does not present a good response with TTNS, PTES may be applied, with equally good responses to the traditional TTNS technique.

This knowledge will help to clarify the role of electrostimulation in the treatment of urgency symptoms and allow evidence-based recommendations on TTNS and PTES as part of the first-line treatment for urgency and diseases such as UUI and OAB syndrome.

### Trial status

The protocol registration was approved on May 17, 2018, and is on version 2 (after the initial evaluation sessions, we computed the sample size calculation and we had to add three more participants, one per group). To date, we have enrolled 52 study participants who have completed treatment, assessments, and follow-up. The initial date of recruitment was June 1, 2018, and the approximate date of recruitment to be completed will be December 2019. Although existing data are being entered, no analysis has yet been performed.

## Data Availability

Not applicable.

## Abbreviations

24 h-VD: 24-Hour Voiding Diary
ANOVA: Analysis of variance
Hz: Hertz
ICIQ-OAB: Incontinence Questionnaire Overactive Bladder
ICT: Informed Consent Term
KHQ: King’s Health Questionnaire
LAMU: Laboratory of Research in Women’s Health
OAB: Overactive bladder
OAB-V8: Overactive Bladder – Validated 8-Question Awareness Tool
PSIS: Posterior superior iliac spine
PTES: Parasacral transcutaneous electrical stimulation
QoL: Quality of life
S2: Second sacral vertebrae
S3: Third sacral vertebrae
TTNS: Transcutaneous tibial nerve stimulation
UFSCar: Federal University of São Carlos
UI: Urinary incontinence
US: Ultrasound
UUI: Urgency urinary incontinence
VAS: Visual analogue scale
μs: Microseconds
